# Supplementary material for: From By-Products to Fertilizer: Chemical Characterization Using UPLC-QToF-MS via Suspect and Non-Target Screening Strategies
Source: Molecules. 2022 May 29;27(11):3498. doi: 10.3390/molecules27113498 (PMC9182003; doi:10.3390/molecules27113498)
Supplement: Supplementary file 1 [file molecules-27-03498-s001.zip › molecules-1733135-supplementary.pdf]

Supplementary Materials

Typical chromatograms of the compounds identified in both raw materials and final products are illustrated in figures S1-S6. Additionally, the temperature change versus time of composting for the onion-based and mushroom-based final products are depicted in figure S7.

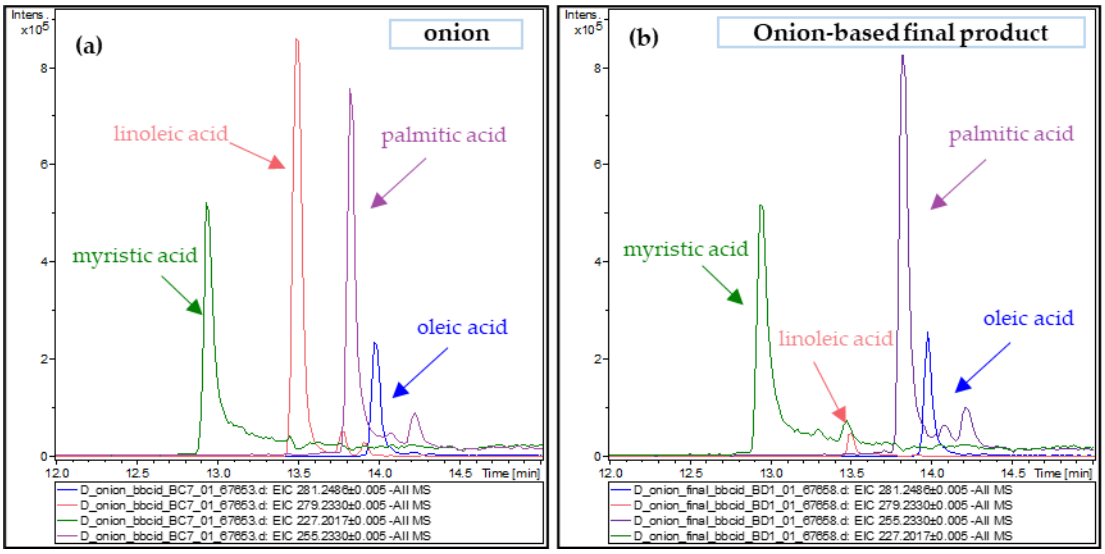

Figure S1: Typical chromatograms of myristic acid (227.2017 ± 0.005 Da), linoleic acid (279.2330 ± 0.005 Da), palmitic acid (255.2330 ± 0.005 Da) and oleic acid (281.2486 ± 0.005 Da) for (a) the onion raw material and (b) the onion-based final product.

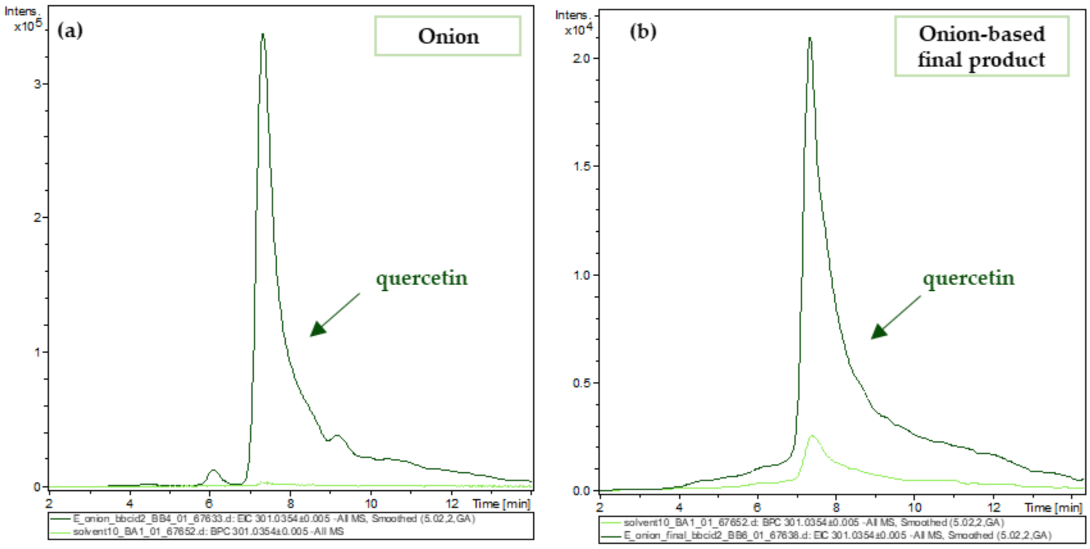

Figure S2: Typical chromatograms of quercetin (301.0354 ± 0.005 Da) for (a) onion raw material and (b) the onion-based final product.

 https://doi.org/10.3390/molecules27113498

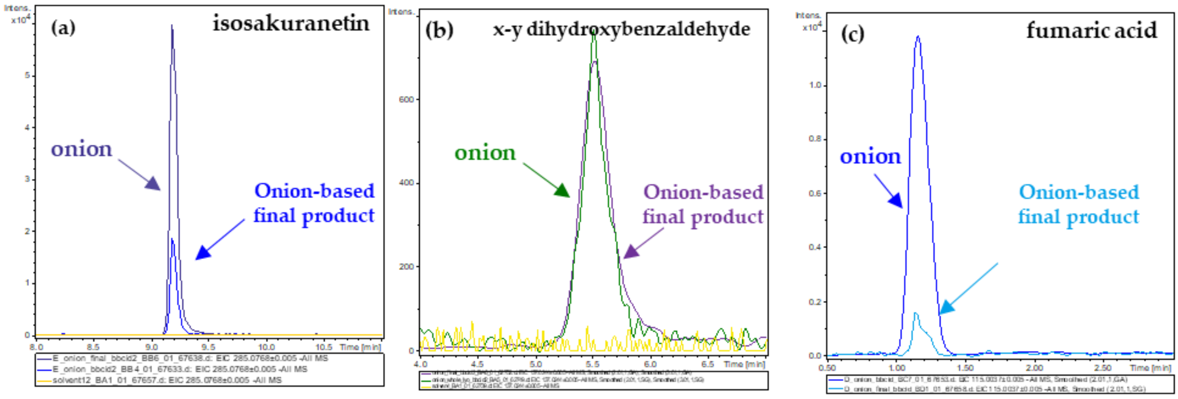

Figure S3: Typical chromatograms of (a) isosakuranetin (285.0768 ± 0.005 Da) (b) x-y dihydroxybenzaldehyde (137.0244 ± 0.005 Da) (c) fumaric acid (115.0037 ± 0.005 Da) for the raw material and its corresponding final product.

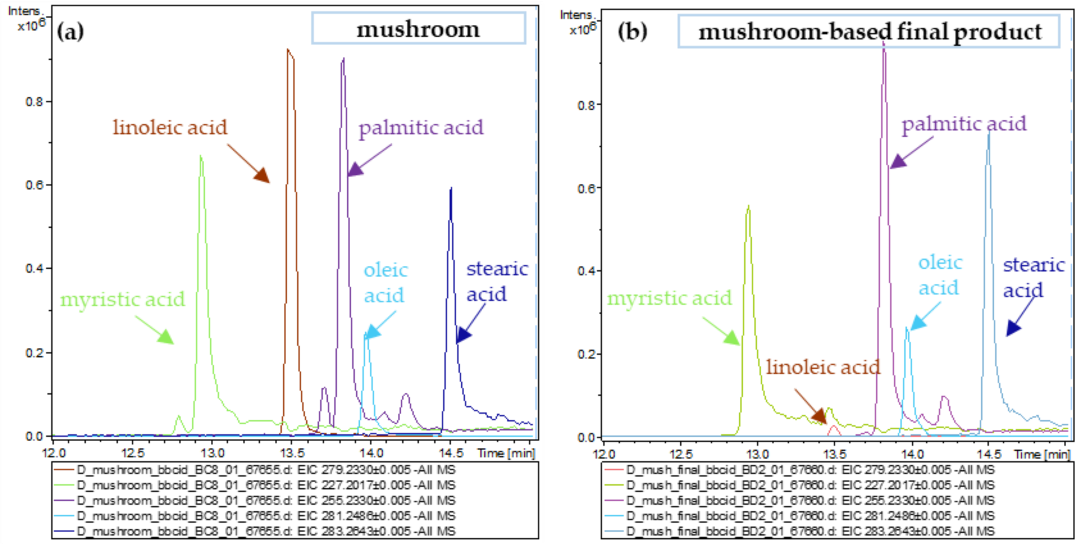

Figure S4: Typical chromatograms of the myristic acid (227.2017 ± 0.005 Da), linoleic acid (279.2330 ± 0,005 Da), palmitic acid (255.2330 ± 0.005 Da), oleic acid (281.2486 ± .005 Da), stearic acid (283.2643 ± 0.005 Da) for (a) the mushroom raw material and (b) mushroom-based final product.

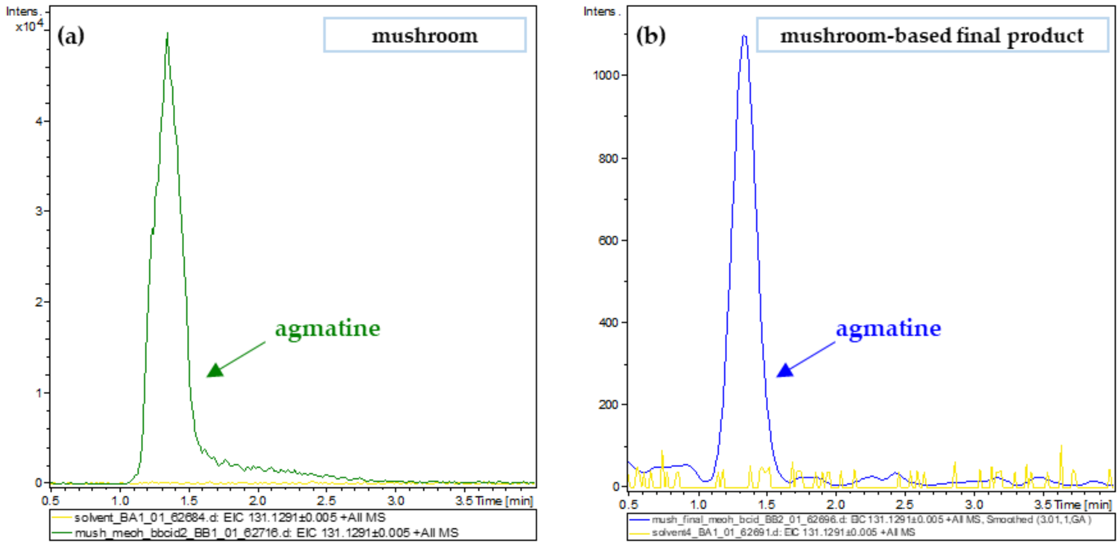

Figure S5: Typical chromatograms of the agmatine (131.1291 ± 0.005 Da) for (a) the mushroom raw material and (b) the mushroom-based final product.

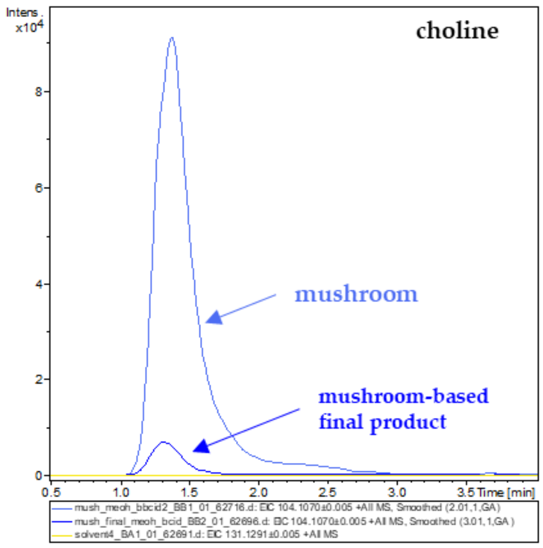

Figure S6: Typical chromatograms of choline (104.1070 ± 0.005 Da) for the mushroom raw material and the mushroom-based final product.

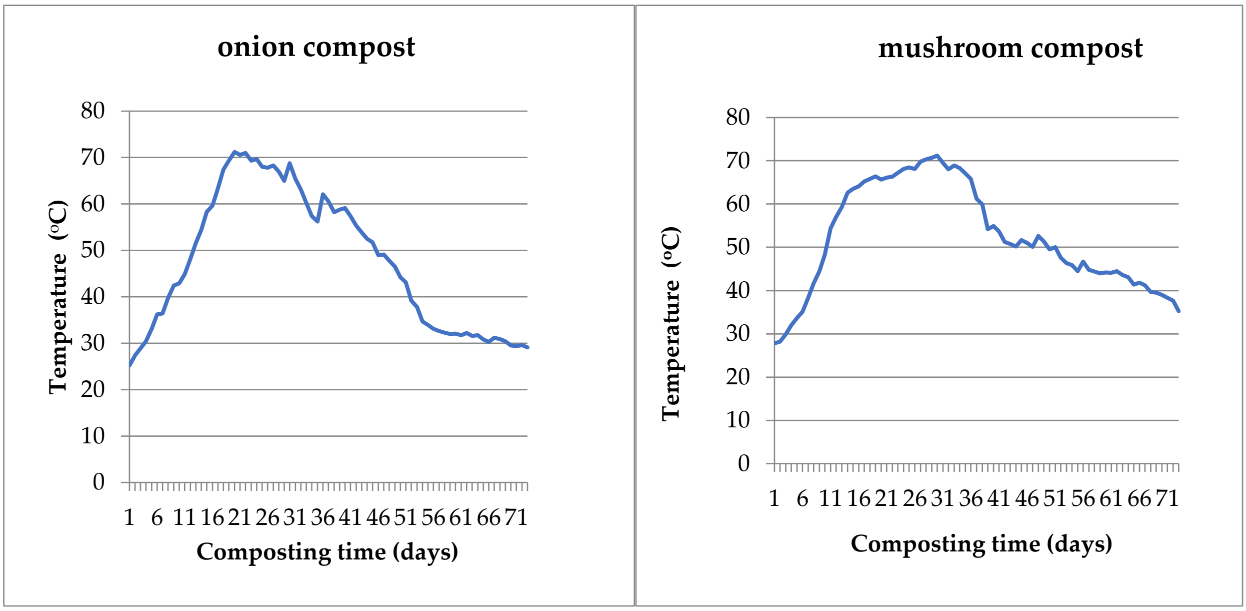

Figure S7. Temperature versus time of composting (a) for the onion-based final product (b) for the mushroom-based final product

The overall results from the analysis of pH, conductivity, nitrogen content, organic carbon, organic matter and the elemental analysis are presented on Table S1.

Table S1. Results of analysis in onion-based and mushroom-based final product.

Parameter	Onion-based final product	Mushroom-based final product
pH	8.44	8.34
Conductivity (mS cm^{-1})	10.1	10.9
Nitrogen (g/100g)	0.0149	0.0029
Organic carbon (g/100g)	0.162	0.256
Organic matter	0.481	0.759
P (mg/kg)	24.1	19.0
Zn (mg/kg)	18.7	18.1
Fe (mg/kg)	14.4	15
Cu (mg/kg)	1.08	0.92
Ni (mg/kg)	0.32	0.32
B (mg/kg)	0.76	<0.2
Mn (mg/kg)	0.72	0.8
Al (mg/kg)	4.36	4.2
Ba (mg/kg)	<0.4	<0.4
Cd (mg/kg)	<0.2	<0.2
V (mg/kg)	<0.2	<0.2
Pb (mg/kg)	<0.4	<0.4
Mo (mg/kg)	<0.2	<0.2
Cr (mg/kg)	<0.04	<0.04
